# Gum katira-silver nanoparticle-based bionanocomposite for the removal of methyl red dye

**DOI:** 10.3389/fchem.2022.959104

**Published:** 2023-01-04

**Authors:** Saruchi, Vaneet Kumar, Diksha Bhatt, Hamed A. El-Serehy, Sadanand Pandey

**Affiliations:** ^1^ Department of Biotechnology, CT Group of Institutions, CT Institute of Pharmaceutical Sciences (CTIPS), Jalandhar, Punjab, India; ^2^ School of Natural Science, CT University, Ludhiana, Punjab, India; ^3^ Department of Zoology, College of Science, King Saud University, Riyadh, Saudi Arabia; ^4^ Department of Chemistry, College of Natural Science, Yeungnam University, Gyeongsan, South Korea

**Keywords:** biopolymer, bionanocomposite, organic contaminates, adsorption, dye

## Abstract

The present study aimed to synthesize gum katira-silver nanoparticle-based bionanocomposite. Different characterization techniques were used to analyze the synthesized bionanocomposite, such as Fourier transform infrared (FTIR) spectroscopy, field emission scanning electron microscopy (FESEM), thermo-gravimetric analysis (TGA), and transmission electronic microscopy (TEM). AgNPs were formed and were 6–20 nm in size. Thermo-gravimetric analysis showed that synthesized nanocomposites are more thermally stable than gum katira. All the reaction conditions, such as time, temperature, pH, solvent, amount of nanoparticles, the concentration of the initiator, crosslinker, and monomer were optimized with respect to swelling. The results showed that the highest percentage swelling (Ps) of Gk-cl-poly(AA) was 796%, and 867% of AgNPs were imbibed by Gk-cl-poly(acrylic acid)-AgNPs. Synthesized bionanocomposite was used as an adsorbent material for the adsorption of methyl red (MR) dye. The effects of different reaction conditions were also optimized to attain maximum adsorption of MR dye. The maximum dye adsorption through Gk-cl-poly(AA)-AgNPs bionanocomposite was 95.7%. Diverse kinetic and isotherm models were used to study the adsorption data. The *R* ^*2*^ value was established as 0.987 and k_2_ was .02671. The greater *R* ^*2*^ value of second-order kinetics over first-order kinetics suggested that MR adsorption by nanocomposite is best explained by pseudo-second-order kinetics, indicating that dye adsorption occurred through chemisorption. The *R* ^2^ value was determined to be .9954. The correlation coefficient values of Gk-cl-poly(AA)-AgNPs were best fitted by the Freundlich adsorption isotherm. Overall, synthesized bionanocomposite is a proficient material for removing of MR dye from wastewater.

## 1 Introduction

Water is precious for all forms of life and different industries must treat it well before discharging it. Owing to advancements in lifestyle, industrial activities have increased at a great pace and the environment has gravely depreciated as a result, particularly the aquatic environment. Toxic organic pollutants from industrial effluents damage the environment, and dyes are the foremost pollutants that contaminate water bodies and are easily recognizable by eye (Gupta, 2009; Okoro et al., 2022). As these dyes have harmful impacts on every type of life, their removal is an urgent matter of concern (Adesina et al., 2015; Saruchi et al., 2020).

There are different types of processes for the removal of harmful dyes from industrial effluents, such as oxidation, electro-coagulation, solvent extraction, trickling filtration, ion exchange, activated sludge, and the use of microorganisms (Wei et al., 2021; Doondani et al., 2022; Sharma et al., 2022). These processes further characterize primary, secondary, and tertiary techniques based on their severity and effectiveness. They are effective but they are non-cooperative in one way or another (Ramaraju et al., 2014; Gisi et al., 2016). Some are effective at high temperatures, and some are not stable at high temperatures, some are effective at low temperatures (same issues with pH), some are costly, some processes have complex methods, and some processes require more labor. Out of all these processes, adsorption has been proven to be the most efficient, cost-effective, and easiest method for removing harmful dyes (Pandey and Mishra, 2013; Pandey and Ramontja, 2016; Aljeboreea et al., 2017; Pandey, 2017; Sharma and Kaur, 2018). The activated carbon obtained from Ficus, bast, etc., has been put to use to study the elimination of methylene blue (MB) dye (Naushad et al., 2016; Naushad et al., 2019; Pathania, 2017).

Nanocomposites are a class of material that includes a minimum of one constituent in the nanometric range. They are labeled as the materials of 21st century due to their unique design and properties, which are different from synthesized materials. They are inventive materials that possess different properties from their constituents (Mahida and Patel, 2016; Seow and Lim, 2016; Sethi and Kaithsaruchi, 2022). Many researchers have worked on the synthesis of nanocomposites. The synthesis of nanocomposites is an outstanding procedure for transforming a hydrogel. These are hydrated polymers with physical or covalent bonds in the presence of particles from the nano range. Currently, certain materials, such as ceramic, polymer, and carbon-based components, as well as metals, are imbibed by or added to the hydrogel matrix to form a matchless nanocomposite hydrogel (Biondi et al., 2015; Mittal et al., 2020a; Saruchi R. et al., 2020). A considerable amount of attention is being paid to the development of a smart nanocomposite material. Such a unique material is important for different fields, such as aerospace, automotive, electronics, and biotechnology industries (Henrique et al., 2009). Additionally, it has a use in the biomedical field, energy storage, construction, and the cosmetic and food industries (Kaith et al., 2012; Mittal et al., 2016; France et al., 2017; Gulia et al., 2022; Jeong, 2022; Pourmadadi et al., 2022).

Double-network hydrogel was prepared with calcium carbonate and polyvinyl alcohol *via* Ca^+2^ crosslinking and a freeze-thaw method. The synthesized material was used as an adsorbent for the removal of the harmful dye MB. Later, the synthesized hydrogel was functionalized with activated carbon, Fe_3_O_4_, and graphene oxide. The Fe_3_O_4_-containing composite is utilized as a heterogeneous Fenton catalyst to degrade MB. The isotherm was best fitted by the Langmuir adsorption isotherm (Naushad and ALOthman, 2015; Kong et al., 2019).

Zhua et al. (2018) synthesized a smart and strong double-network hydrogel, in which the first network comprised PEG merged with poly (acryloyloxyethyltrimethyl ammonium chloride) and the second network comprised acrylic acid, acrylamide, and MBA. This double-network hydrogel has great potential in biomedical applications. A poly (vinyl alcohol)- and poly (acrylic acid)-based hydrogel was formulated and customized to a nanocomposite material by absorption of montmorillonite by the hydrogel matrix aided by gamma irradiation (Mohsen et al., 2017; Thombare et al., 2018). A poly acrylic acid- and poly acrylamide-based mechanico-chemical and pH responsive co-polymeric accusative system has been formulated to permit the antagonistic faction of the solution. The system was inspired by a biologically based accusative system exhibiting some swelling and contraction in response to changes in external stimuli (Zarzar et al., 2011). Acrylamide and acrylic acid-based tubular-shaped co-polymer hydrogels have also been prepared by Nesrinne and Djamel (2017) with the help of free radical polymerization using distilled water. Ammonium persulfate (APS) and tetramethyl ethylenediamine (TEMED) were used as an initiator system (Rana et al., 2021a; Rana et al., 2021b).

AgNPs mediated toxicity, even in deficient concentrations, and their harmful biological consequences when present in water. The amount of AgNPs released in the effluent water, sludge, and aquatic environment is of significant concern. Experiments with AgNPs have achieved immense success. AgNPs are one of the most valuable metal nanoparticles. These AgNPs are personalized to different materials, such as graphene, metal, fiber, and polymers, to form a silver-based composite that improves electrical conductivity, chemical and thermal stability, adsorption capacity photocatalysis, and other capabilities. These nanocomposites can be applied to medicine, the environment, industry, biology, food, and other fields (Wu et al., 2021). Thus, in the present study, silver nanoparticle-embedded gum katira-based bionanocomposite was synthesized. Synthesized bionanocomposite was characterized using different techniques, such as FTIR, SEM, and TEM. The main objective of the present study was to synthesize eco-friendly bionanocomposite that can be used as an adsorbent for the exclusion of the harmful dye methyl red.

## 2 Experiments

### 2.1 Materials and method

Gum katira (Gk) (backbone), Ammonium persulfate (APS) (initiator), N,N′– methylenebisacrylamide (MBA) (crosslinker), acrylic acid (AA) (monomer), and silver nitrate were procured from Loba Chemie Pvt Ltd., Mumbai, India. All chemicals were used as received. All solutions were prepared in double-distilled water.

### 2.2 Synthesis of Gk-cl-poly(AA)

In a typical experiment, 1 g of gum katira was dissolved in 10 ml of deionized water at ambient temperature to homogenously dissolve the biopolymer. The required amount of acrylic acid was added and stirred for 1 h. Then, the required amounts of MBA and initiator APS were added. A specific amount of ammonium persulfate, as well as MBA, was added to the reaction mixture and continuously stirred until the mixture was uniform and homogeneous. The reaction mixture was then warmed to the required temperature, stirred for a specific amount of time, and poured into acetone, and the precipitated grafted guar gum was isolated by filtration. The mixture was then washed twice with acetone, filtered, and dried at 60°C in an oven until it reached a constant weight. Every single reaction parameter, such as time, temperature, pH, solvent, initiator, crosslinker, and monomer, was optimized with respect to the percentage of swelling (Ps). The homopolymer that formed was separated by extraction with deionized water, and then Gk-cl-poly(AA) was dried (Saruchi K. V. and Kumar, 2019; Saruchi T. P. and Kumar, 2019).

### 2.3 Synthesis of AgNP-Embedded Gk-cl-poly(AA)

Gk-cl-poy(AA) (1 g) was solubilized in 10 ml of water; .1 M NaOH solution was used to maintain a pH of 10. Silver nitrate (1 mmol) solution was added slowly with stirring and the mixture was then heated for 5 min. The color of the solution immediately changed to brownish-yellow, which confirmed the development of AgNPs. Gk-cl-poly(AA)-AgNP bionanocomposite was taken out, washed, and dried until a constant weight was obtained (Sethi et al., 2022).

### 2.4 Swelling studies

The swelling of synthesized Gk-cl-poly(AA) and Gk-cl-poly(AA)AgNPs was studied. A specific amount of synthesized Gk-cl-poly(AA) and Gk-cl-poly(AA)AgNPs was submerged in 100 ml of deionized water. Then, after a certain period of time, Gk-cl-poly(AA) was removed, wiped softly, and weighed. The percentage of swelling (Ps) in swollen Gk-cl-poly(AA) was determined using the following equation (Kaith et al., 2010; Mittal et al., 2020b):

$$P_{s}=\frac{W_{s}-W_{d}}{W_{d}}\;X\;100$$

where, W_s_ and W_d_ are the weight of the swollen and dried sample, respectively.

### 2.5 Instrument details

FTIR spectra were recorded with a Bruker Alpha FTIR spectrometer (Bruker FTIR SPECTROMTR, Model APHA, Germany) using KBr pellets (range 4,000–400 cm^−1^). The morphological variations were studied using a high-resolution SEM (JEOL S150A, Japan). Samples were dried and gold plated for SEM characterization. The thermal behavior of the samples was studied using TGA/DTA 6300 and SII EXSTAR 6000 instruments (Inkarp Instruments, Japan). TGA scans were recorded from 50°C to 700°C at a heating rate of 10°C per minute. Nanoparticle sizes were investigated using a transmission electron microscopy (TEM) (JEOL-JEM-2100, Japan). TEM samples were prepared on a carbon film supported by a copper grid.

## 3 Results and discussion

### 3.1 Characterization

Synthesized bionanocomposite was characterized using different techniques, such as FTIR, SEM, and TEM, and details of the results from this are as follows:

#### 3.1.1 FTIR

**FIGURE 1:**
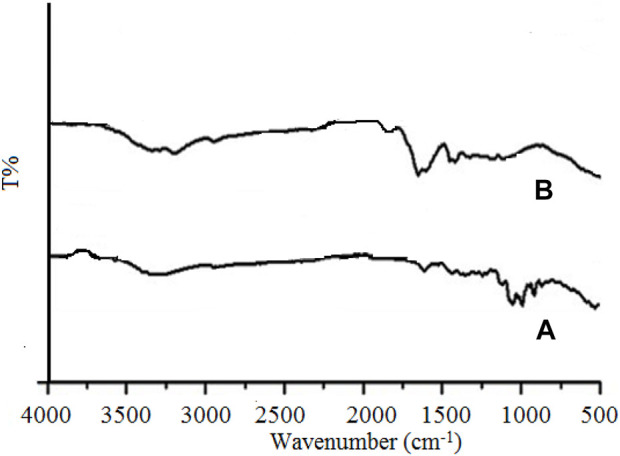
**(A,B)** FTIR spectra of gum katira **(A)** and Gk-cl-poly(AA) **(B)**.

The FTIR of Gk and Gk-cl-poly(AA) was analyzed using a PerkinElmer spectrophotometer. Figure 1A depicts the wider peaks noted in the IR spectrum of gum katira at 3,325 cm^−1^ due to O-H stretching of the intermolecular hydrogen-bonded OH group, at 2,915 cm^−1^ because of C-H stretching, at 1,667 cm^−1^ due to the carbonyl group, at 1,345 cm^−1^ due to C-O stretching of °3 alcohol, and at 1,237 cm^−1^ due to C-C deformation in the methyl group. The IR spectrum of Gk-cl-poly(AA) (Figure 1B) illustrates additional peaks at 2,931, 2,659, 1,699, and 1,416 cm^−1^ due to C-H stretching, O-H of COOH, C=O stretching of aryl carboxylic acid, and C-H group and C-C stretching of the C-C group, respectively. The biological constituents that contain these functional groups in Gk-cl-poly(AA) are acrylic acid and MBA. It is clear from the FTIR results that grafting and crosslinking occurred in the backbone and monomer.

#### 3.1.2 FESEM

**FIGURE 2:**
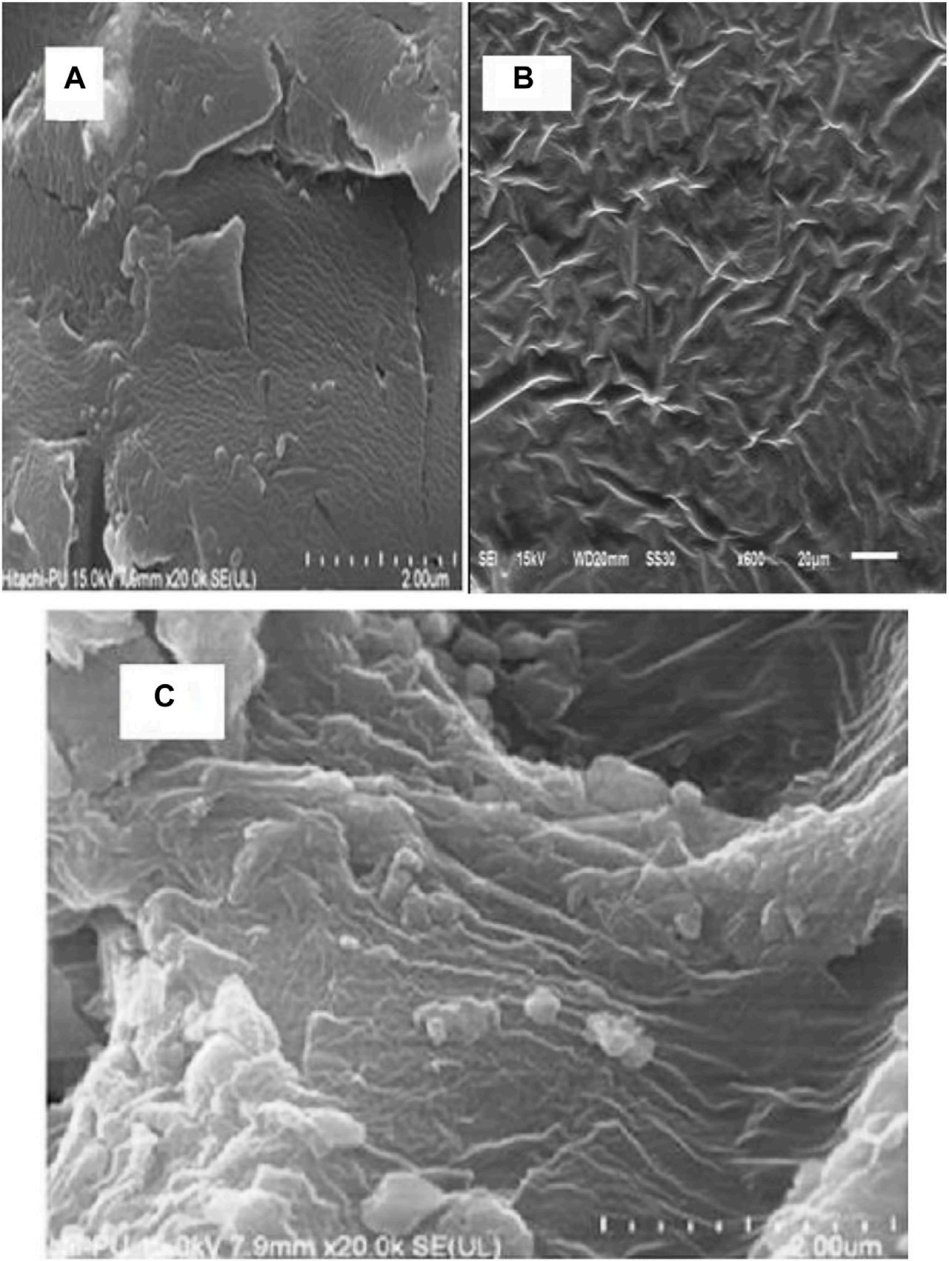
**(A–C)**. FESEM images of gum katira **(A)** and Gk-cl-poly(AA) and Gk-cl-poly(AA)AgNPs **(B)**.

The exterior morphology of Gk, Gk-cl-poly(AA), and Gk-cl-poly(AA)-AgNPs was examined by SEM. A smooth surface was observed in gum katira (Figure 2A). The surface morphology of Gk did not feature ridges, grooves, etc. For Gk-cl-poly(AA), a rough surface with pits was observed, which was a result of crosslinking and grafting (Figure 2B). FESEM of synthesized Gk-cl-poly(AA)AgNPs revealed a structure with well-distributed AgNPs (Figure 2C). This confirmed that AgNPs are imbibed by the Gk-cl-poly(AA).

#### 3.1.3 Thermogravimetric analysis (TGA)

TGA evaluates the mass of Gk, Gk-cl-poly(AA), and Gk-cl-poly(AA)-AgNPs, while heating, cooling, or a stable temperature represents the stoichiometric decomposition of components and the mass of substance missing at different steps. Combustion products generated by burning of the samples escape into the atmosphere and the mass of the substance decreases leaving behind ash.

**FIGURE 3:**
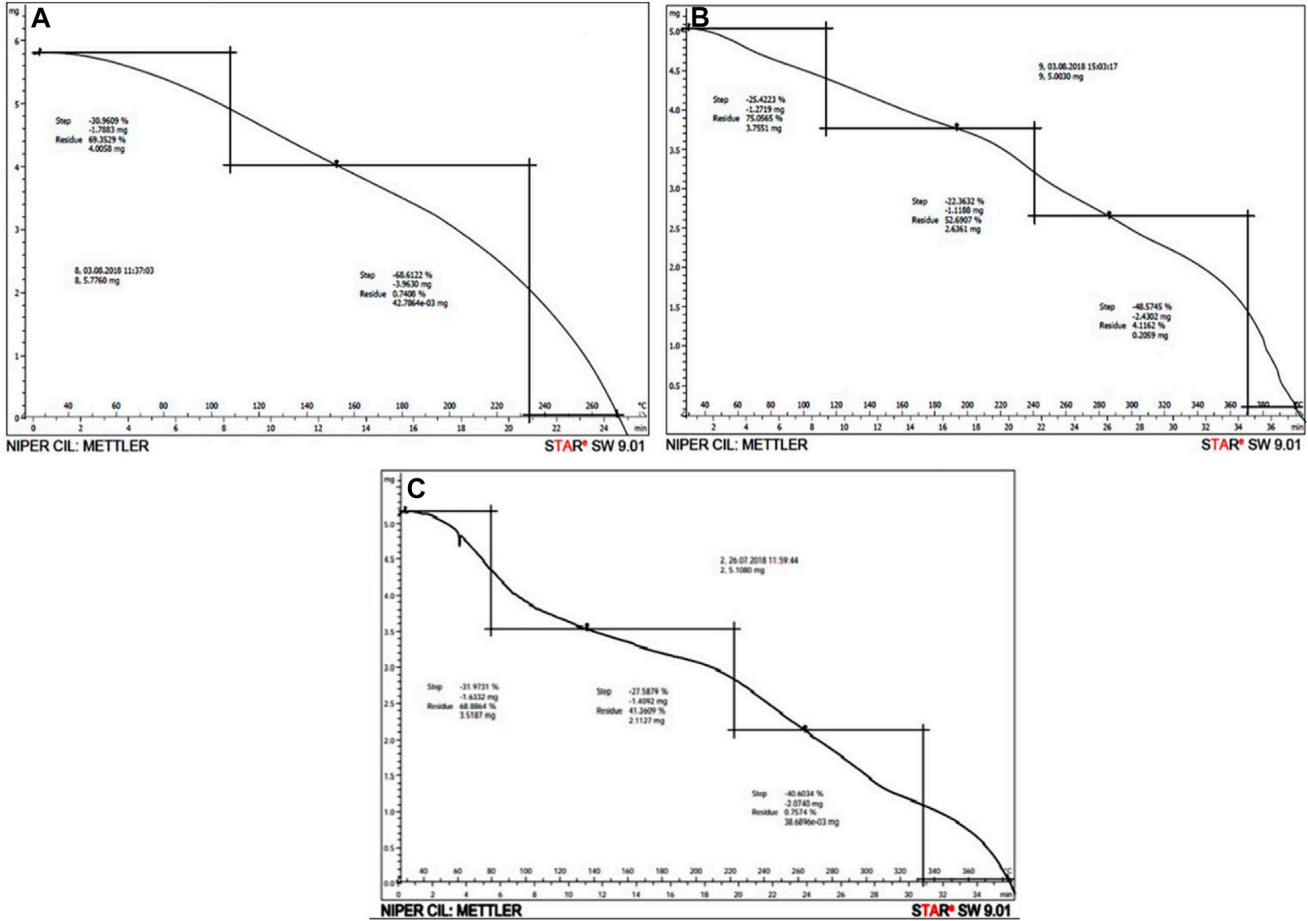
**(A–C)** TGA of gum katira **(A)**, Gk-cl-poly(AA) **(B)**, and Gk-cl-poly(AA)-AgNP nanocomposite **(C)**.

Gk was heated up to 270°C. The thermal stability of SIPN was observed up to 105°C, at which point volatile substituents were lost. In the second step, further pyrolysis took place up to 235°C, at which point the decomposition process caused the release of carbon monoxide and carbon dioxide. Once temperatures rose above 235°C, a black residue was left behind. Polymer content weight loss during the different steps was as follows: 30.9% during the first step and 68.6% during the second step (Figure 3A).

As depicted in Figure 3B, the disintegration of Gk-cl-poly(AA) occurred up to a temperature of 390°C. During the primary step of burning of Gk-cl-poly(AA), volatile components were lost up to a temperature of 110°C. The secondary step featured pyrolysis of the polymer up to a temperature of 240°C, at which point breakdown occurred, resulting in the release of carbon monoxide. During the third step, further incineration of carbon occurred up to a temperature of 370°C. A few inorganic components were left as a residue at 390°C. Weight loss of Gk-cl-poly(AA) during pyrolysis was as follows: 25.4% during the first step, 22.4% during the second step, and 48% during the third step (Figure 3B).

The decomposition of Gk-cl-poly(AA)-AgNPs occurred at 380°C. During the first step, sample burning was accompanied by the escape of some volatile components up to 80°C. During the second step, heating continued up to 220°C, further advancing the decomposition process. During the third step, the combustion of carbon took place up to a temperature of 330°C. At 330°C, the combustion of carbon black additives took place. Some amount of residue was left behind at 380°C. The loss of weight of the sample during thermal analysis was 31.97% during the first step, 27.58% during the second step, and 40.60% during the third step (Figure 3C). These results showed that thermal stability was enhanced by imbibing synthesized AgNPs onto Gk-cl-poly(AA) (Vellora et al., 2015; Thakur et al., 2017).

#### 3.1.4 Transmission electronic microscopy (TEM)

**FIGURE 4:**
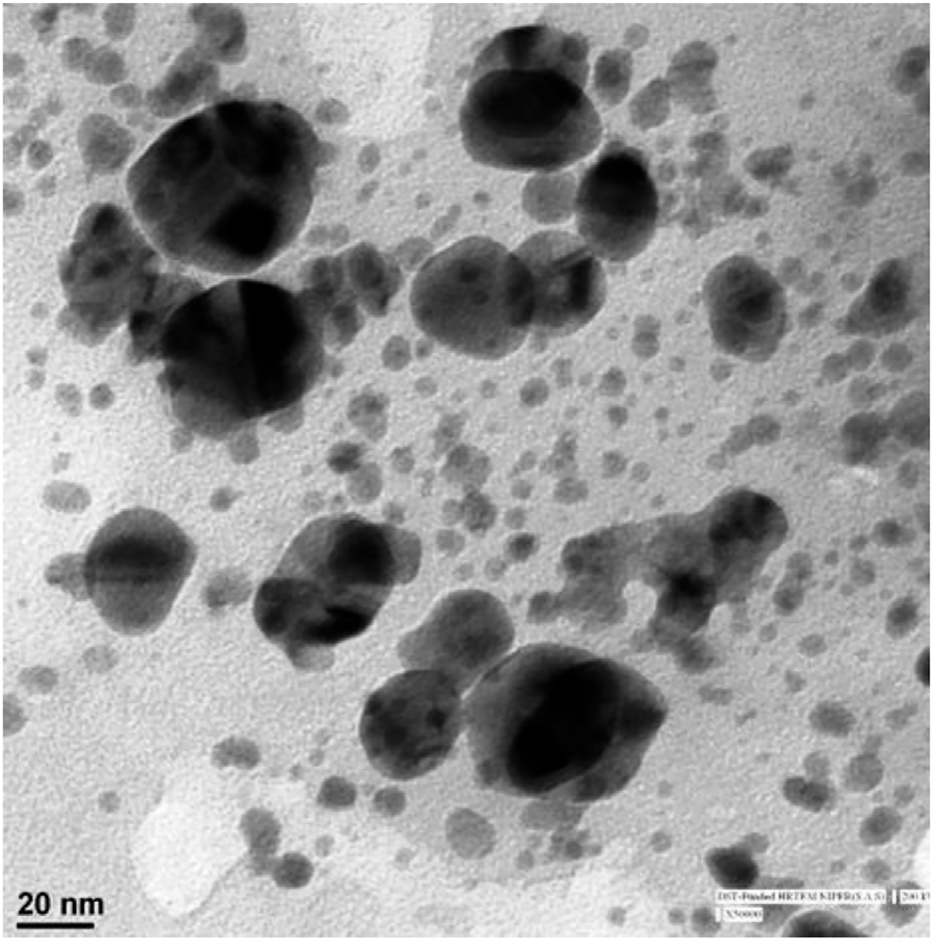
TEM image of synthesized silver nanoparticles.

Particle size and shape was analyzed by TEM, which allowed the distinctiveness of very small-sized samples to be observed. The synthesized AgNPs were nanosized and clearly spherical and ellipsoidal in shape (Figure 4).

### 3.2 Optimization of various reaction conditions in Gk-cl-poly(AA) synthesis

#### 3.2.1 Impact of reaction time

**FIGURE 5:**
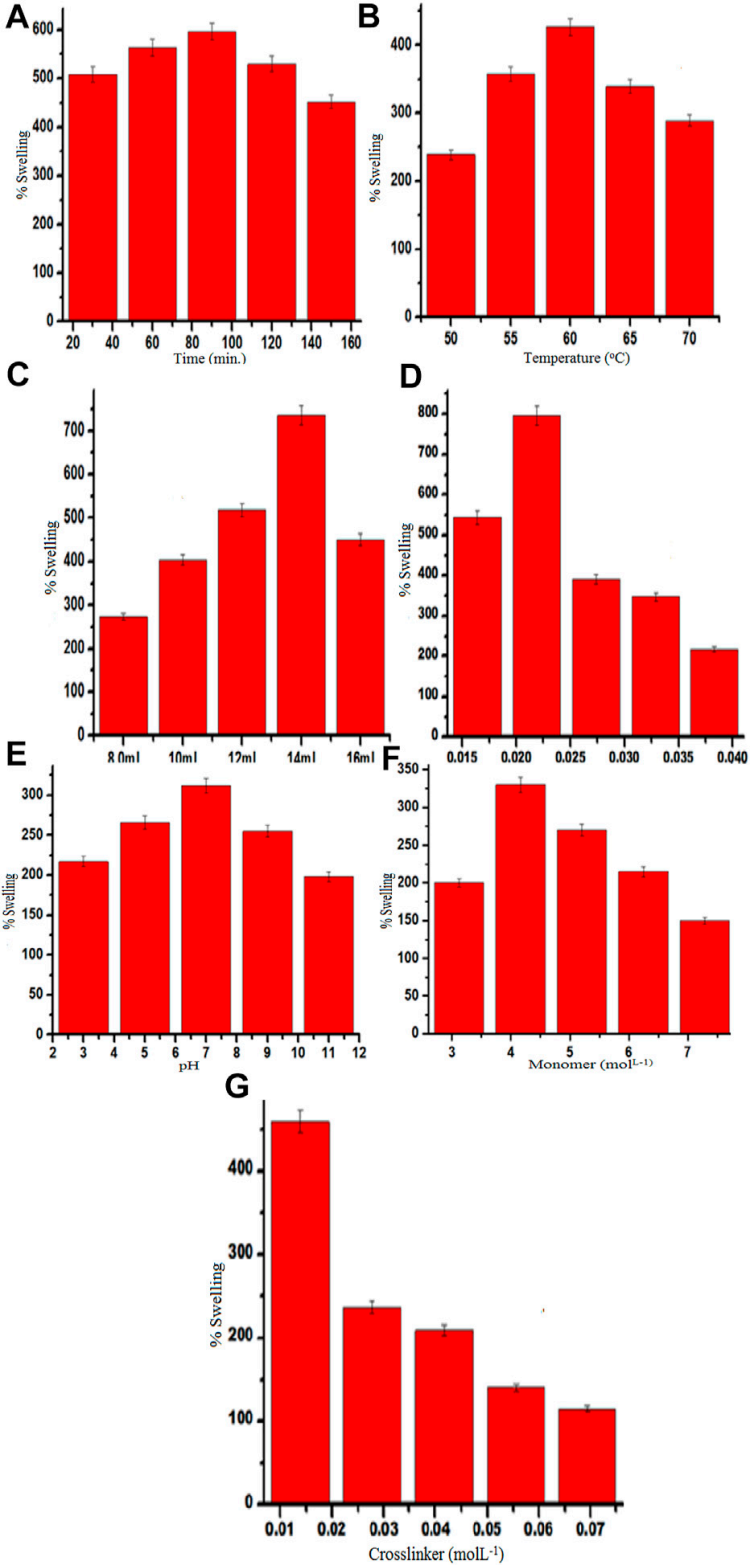
**(A–G)** Effect of time **(A)**, temperature **(B)**, solvent **(C)**, initiator concentration **(D)**, pH **(E)**, monomer concentration **(F)**, and crosslinker concentration **(G)** on the Ps of Gk-cl-poly(AA).

Ps increases as reaction time increases and a maximum swelling of 596% (Figure 5A) was attained after 90 min, but swelling decreased beyond this point. This result might be due to the time period extending beyond the optimal time and/or the porous network of the polymer becoming fully saturated and thus becoming unable to absorb any more solvent. Additionally, it may be caused by the dominance of homopolymerization over graft co-polymerization with an increase in time. All these factors may be responsible for a diminishing Ps with time (Naushad, 2014; Saruchi and Kaith, 2016).

#### 3.2.2 Impact of reaction temperature

The impact of temperature was investigated as it is an important parameter for grafting. The highest Ps was obtained at 60°C (426%) (Figure 5B). When the temperature was increased beyond the optimal level, Ps decreased due to the polymeric matrix becoming more flexible, leading to the breakdown of secondary interaction. Higher temperatures lead to the collapse of the polymeric chains, which causes desorption, and as a result of this, swelling decreases (Ting and Zhiyuan, 2018)^.^

#### 3.2.3 Impact of pH

The highest Ps was obtained at a neutral pH (312%) (Figure 5C). There are ample hydrogen ions at this level, which protonates the functional group, repelling the polymeric chains and thus increasing swelling. While in an alkaline medium, de-protonation of the functional groups occurs, and as a result, repulsions inbetween the polymeric chains decrease, thus decreasing Ps (Wang et al., 2015; Zakaria et al., 2017).

#### 3.2.4 Impact of APS concentration

Ps increased with an increase in APS concentration, and optimum Ps (796%) (Figure 5D) was obtained at .022 mol/L of APS. This result can be explained by the abundance of active free radicals at the start of the reaction that enhance grafting; however, as maximum grafting is reached, increases in APS concentration beyond optimal levels lead to the termination of the reaction and also homopolymerization, thus decreasing Ps due to the excess production of hydroxyl and sulfate ions, resulting in early cessation of the reaction.

#### 3.2.5 Impact of the amount of solvent

Ps increased as the amount of solvent increased, and a maximum Ps of 736% was attained using 14 ml of solvent (Figure 5E). The concentration of OH-ions was adequate to promulgate a polymerization reaction up to the optimal level of solvent; however, further increases in the amount of solvent cause the concentration of OH^−^ to markedly increase to a level that terminates the polymerization reaction, thus decreasing Ps (Saruchi et al., 2014).

#### 3.2.6 Impact of monomer concentration

The highest Ps (318%) was obtained at a monomer concentration of 4.1632 molL^−1^ (Figure 5F). Ps decreased when monomer concentrations were above the optimal level. Initially, the availability of large numbers of functional groups increases co-polymerization; however, AA concentrations above the optimal level lead to the formation of homopolymer, rather than graft co-polymer, thus decreasing Ps.

#### 3.2.7 Impact of MBA concentration

The highest Ps (459%) was observed with .0139 molL^−1^ of MBA (Figure 5G). Ps increases initially because of the formation of crosslinks inbetween the Gk and AA chains. However, when MBA concentration exceeds the optimal level, Ps decreases because the higher concentration leads to the structure becoming compacted.

#### 3.2.8 Optimization of the amount of AgNPs in Gk-cl-poly(AA)-AgNPs bionanocomposite

**FIGURE 6:**
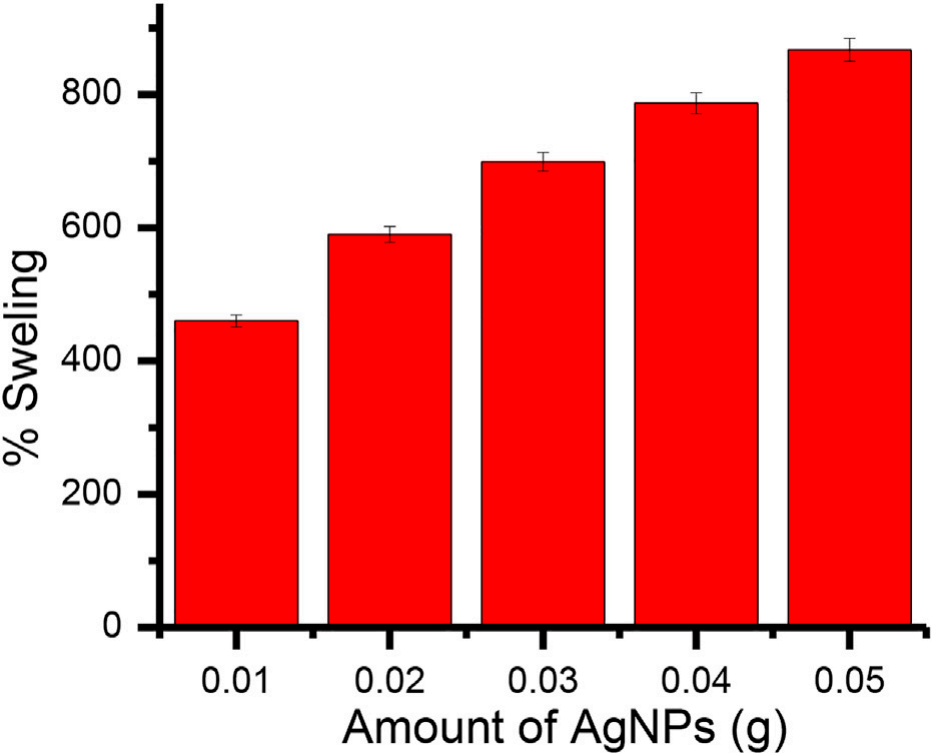
The impact of AgNPs on the swelling of Gk-cl-poly(AA)AgNP bionanocomposite.

The amount of nanoparticles (AgNPs) added during the preparation of Gk-cl-poly(AA)-AgNP bionanocomposite varied from .01 to .05 g. The percentage of swelling increased from .01 to .03 g and then decreased at higher levels, exhibiting a maximum swelling of 867% at .03 g of nanoparticles (Figure 6).

### 3.3 Adsorption of methyl red (MR) dye

#### 3.3.1 Impact of contact time

**FIGURE 7:**
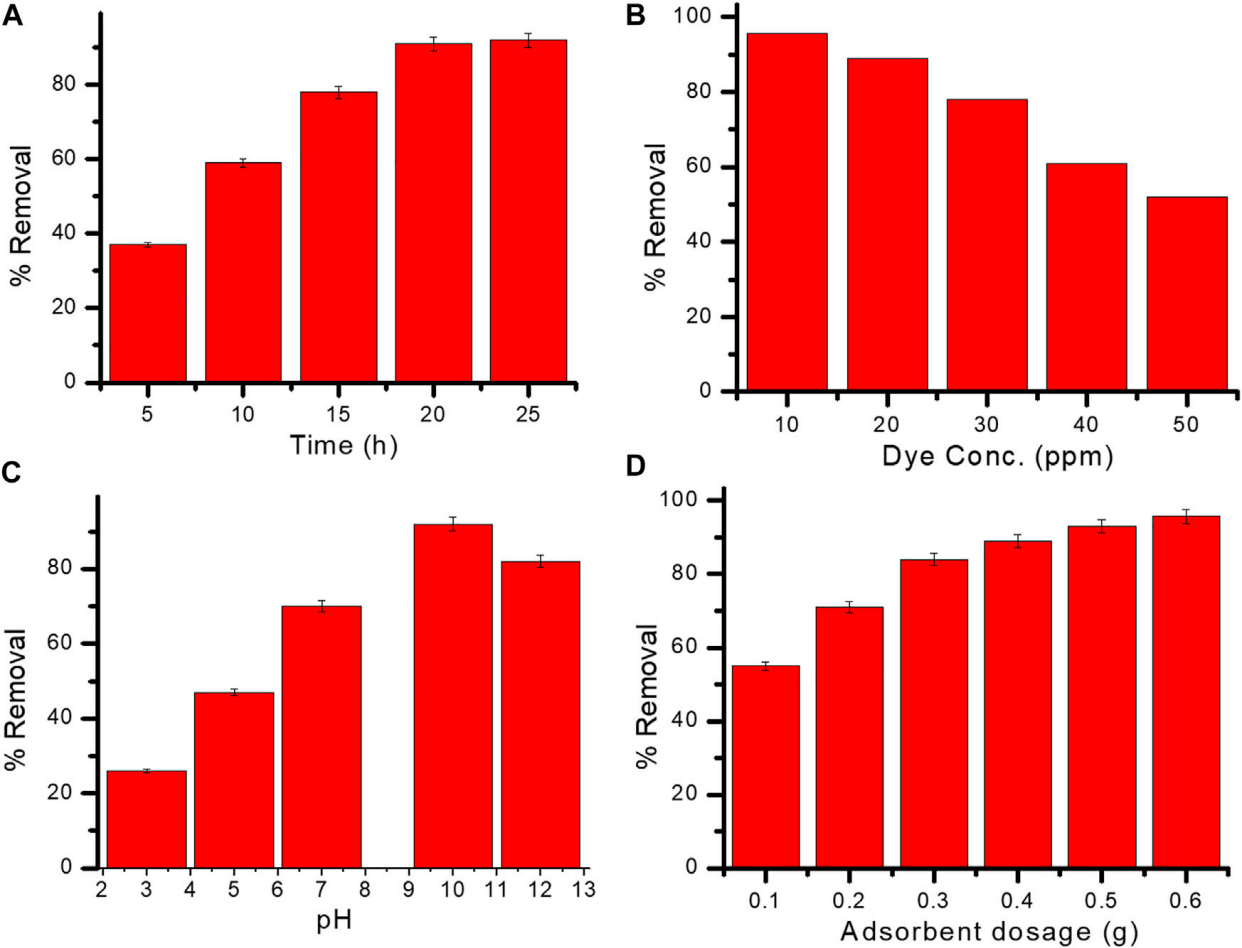
**(A–D)** Effect of contact time **(A)**, dye concentration **(B)**, pH **(C)**, and adsorbent dosage **(D)** on the adsorption of methyl red dye onto Gk-cl-poly(AA)-AgNP bionanocomposite.

MR dye removal was slow at the early stage, but with time, dye removal increased. An equilibrium was attained at 20 h, at which point dye removal was at its maximum (92%) (Figure 7A). Primarily, the dye solution’s concentration gradient was high; furthermore, there is a vacancy at the site of the adsorbent. Readiness of the adsorption sites increase the adsorption rate (after 5 h) but afterwards adsorption diminishes, resulting in the formation of a monolayer of MR dye over the surface of the adsorbent.

#### 3.3.2 Impact of dye concentration

The initial concentration of the dye had a significant impact on their eradication; therefore, differing concentrations of dye solution (10–50 ppm) were examined with respect to the percentage of dye removal. The highest percentage of MR dye removal was 95.6% at 10 ppm (Figure 7B); higher concentrations of dye led to decreased removal due to saturation of the active sites of the adsorbent (Saruchi et al., 2018).

#### 3.3.3 Impact of pH

pH is an important factor in the chemistry between the adsorbent surface and the dye molecule. Electrostatic interaction takes place between adsorbate and adsorbent. Dye extraction increased as pH increased, and equilibrium was attained at pH 10. No further increases in the adsorption of dye were observed beyond pH 10 due to occupancy of active sites. The highest percentage of dye removal (92%) occurred at pH 10 (Figure 7C).

#### 3.3.4 Impact of adsorbent dosage on the removal of dye

The outcome of formulated Gk-cl-poly(AA)-AgNP dose on MR dye removal was examined at a range of doses (.1–0.6 g) (Figure 7D). The adsorption of dye increased as dosage level increased, perhaps due to the increased exposure of surface area of the sorbent and vacancy of active sites intended for adsorbent. The highest level of dye removal (95.7%) was observed at a dosage level of .6 g.

### 3.4 Adsorption kinetics of MR dye onto Gk-cl-poly(AA)-AgNP bionanocomposite

Adsorption kinetics play an important role in the relationship between the time required for adsorption and the dye molecule exclusion rate. Kinetic studies were carried out at various time intervals using pseudo-first- and pseudo-second-order kinetics. Result showed that initially there is an increase in dye uptake but eventually an equilibrium is reached, after which dye uptake decreases. The abundance of adsorption sites accounts for the initial dye adsorption, but later these active sites become exhausted, and thus, a constancy is reached (Naushad et al., 2015; Malakootian and Heidari, 2018; Chowdhury et al., 2019).

**FIGURE 8:**
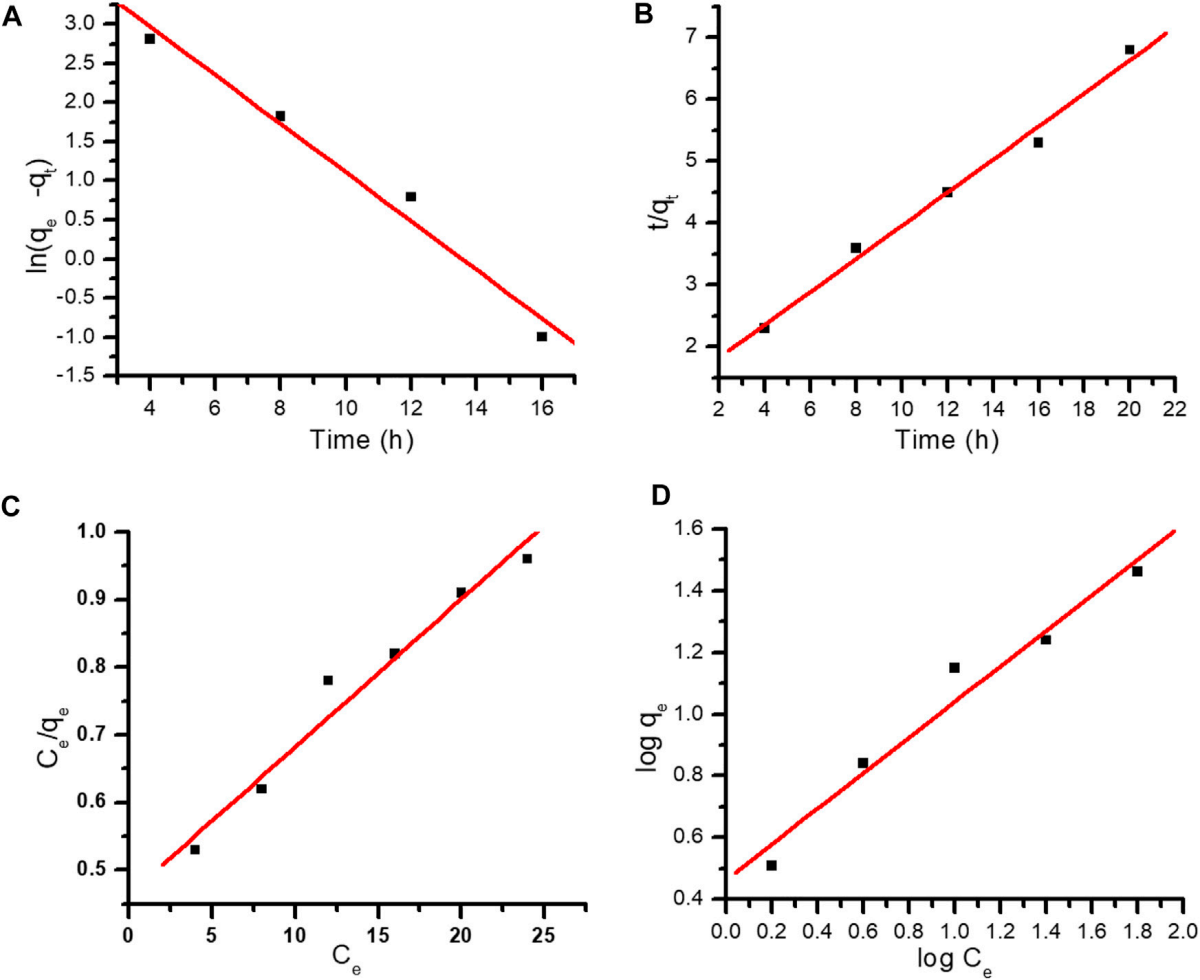
**(A)** Pseudo-first-order kinetics. **(B)** Pseudo-second-order kinetics. **(C)** Langmuir adsorption isotherm. **(D)** Freundlich adsorption isotherm.

Pseudo-first-order kinetics are expressed as:

$$\ln\left(q_{e}-q_{t}\right)=\ln q_{e}^{-k1t}$$

where qe is the equilibrium adsorption capacity (mg g^−1^), q_t_ is the adsorption capacity at time t (mg g^−1^), and k1 refers to the pseudo-first-order rate constant (g mg^−1^min^−1^) (Imaga and Abia., 2015). Log q_e_-q_t_ was plotted against different time intervals. The value of k was attained from the slope. The *R* ^*2*^ value was very low, i.e., .84278, and k1 was determined to be .29087, and pseudo-first-order kinetics did not fit into the experimental data (Figure 8A).

Similarly, second-order kinetics was applied using the equation:

$$\frac{t}{q_{t}}=\frac{1}{K_{2}q_{e}^{2}}+\left(\frac{1}{q_{e}}\right)t$$

where q_e_ and q_t_ refer to the amount of dye adsorbed at equilibrium and at time t, whereas k_2_ represents the second-order rate constant (Figure 8B).

### 3.5 Adsorption isotherms

The Freundlich and Langmuir adsorption isotherms were applied to study the equilibrium isotherms. The *R* ^2^ values of both the models were compared.

The Langmuir equation is expressed as:

$$q_{e}=\frac{Q_{m}bC_{e}}{1+bC_{e}}$$

where, C_e_ (mg L^−1^) is the equilibrium concentration of MR dye, q_e_ represents the quantity of adsorbed dye molecules at equilibrium (mg g^−1^), b is the Langmuir constant, and Q_m_ is the monolayer capacity. The values of b and Q_m_ were determined from the intercept and slope from the plot of 1/q_e_ vs. C_e_. The *R* ^*2*^ value was .7184 (Figure 8C). The Langmuir model did not fit the experimental data so the Freundlich adsorption isotherm was applied (Thakur et al., 2016; Silva et al., 2019).

The Freundlich adsorption isotherm is represented as:

$$\ln q_{e}=\ln k_{f}+\left(\frac{1}{n}\right)\ln C_{e}$$

where, q_e_ refers to the amount of dye adsorbed, Ce corresponds to the equilibrium concentration of MR in solution, k_f_ refers to the Freundlich constant, and 1/n refers to the adsorption capacity. The values of k_f_ and *n* can be determined from the intercept and slope (Wang et al., 2018; Saruchi et al., 2022). The correlation coefficient was best fitted in the Freundlich adsorption isotherm. *R* ^2^ was determined as .9954, which is closer to unity than that observed with the Langmuir model (Figure 8D). Kinetic and isotherm parameters for the adsorption of MR over synthesized nanocomposite are shown in Tables 1, 2, respectively.

**TABLE 1:** Kinetic parameters for the adsorption of methyl red over synthesized bionanocomposite.

Model	Kinetic parameter	
Pseudo first order	K_1_ × 10^–2^ (min.^−1^)	.29087
	q_e_ (mg g^−1^)	3.92758
	q_e_ exp_._ (mg g^−1^)	3.71
	*R* ^2^	.84278
Pseudo second order	K_2_ (g mg^−1^ min.^−1^)	.02671
	q_e_ (mg g^−1^)	.14697
	q_e_ exp_._ (mg g^−1^)	.15
	*R* ^2^	.98783

**TABLE 2:** Isotherm parameters for the adsorption of methyl red over synthesized bionanocomposite.

Isotherm model	Isotherm constant	
Langmuir	Q _m_	.635
	B	.7213
	*R* ^2^	.7184
	R_L_	.3059
Freundlich	1/n	.8156
	k_f_	2.320
	*R* ^2^	.9954

### 3.6 Regeneration study

When the stability and recycling of the synthesized bionanocomposite was inspected using ethanol at low concentration (1%–5%), the regeneration efficiency was 88.6%, 79.3%, 68%, and 53% in the first, second, third, and fourth cycles, respectively. It was clear from the results that synthesized bionanocomposite was stable and retained its adsorption capacity for longer. Therefore, synthesized bionanocomposite is an effective and efficient adsorbent for water treatment (Hosny et al., 2022a).

### 3.7 Leaching studies

The stability of the synthesized AgNPs in the Gk-cl-poly(AA) was investigated by immersing .2 g of Gk-cl-poly(AA)AgNP bionanocomposite in 20 ml of distilled water, which was then vigorously shaken in a water-bath shaker (35°C, 250 rpm) for different time periods. After a specific period of time, the sample was checked for the quantity of AgNPs leached into the water using a UV-Vis spectrophotometer at 432 nm. It was clear from the results that AgNPs leached into the water in very small amounts (.1 μg/L) after 1 h and increased to .35 and .42 μg/L after 4 and 12 h, respectively. The acceptable concentration for Ag in drinking water, according to the WHO, is 8–49 μg/L. Thus, it can be concluded from the above results that it is safe to use the bionanocomposite as an adsorbent.

### 3.8 Comparison with other adsorbents

The efficiency of synthesized bionanocomposite was compared with the published literature. Green synthesizes of bimetallic Ag/ZnO@biochar nanocomposite showed a photodegradation efficiency of up to 70.3% (Hosny et al., 2022). Biogenic Ag@biochal nanocomposite showed a maximum degradation of methylene blue of up to 88.4% (Eltaweil et al., 2022). CMC-g-PAM superporous monoliths were used for the adsorption of methyl violet and methylene blue and showed a maximum adsorption of up to 92.1% and 93.5%, respectively (Wang et al., 2017). Zero-valent iron-supported lemon-derived biochar was used for the adsorption of methylene blue and its maximum adsorption capacity was 195.4 mg/g (Abd El-Monaem et al., 2022). Sulfonated graphene oxide impregnated with cellulose acetate showed a maximum adsorption capacity of 234.74 mg/g for methylene blue dye (Basha et al., 2022). It is clear from the literature that synthesized bionanocomposite is an efficient adsorbent of dye.

## 4 Conclusion

The present study demonstrated the synthesis of a novel gum katira and a silver nanoparticle-based bionanocomposite. TEM images confirmed the formation of silver nanoparticles. FTIR and SEM confirmed that grafting occurred in the gum katira and acrylic acid. Grafting and crosslinking made the synthesized bionanocomposite thermally stable. The maximum percentage swelling of Gk-cl-poly(AA) and Gk-cl-poly(AA)-AgNPs was 796% and 867%, respectively. The highest dye adsorption from Gk-cl-poly(AA)-AgNPs was 95.6%. The *R* ^2^ and k^2^ values were .98783 and .02671, respectively. The greater *R* ^*2*^ value of second-order kinetics compared with first-order kinetics suggested that MR adsorption by nanocomposite is best explained by pseudo^-^second-order kinetics. The foregoing discussion has shown that Gk-cl-poly(AA)-AgNPs are an effective adsorbent for the removal of the harmful dye MR and is eco-friendly in nature. This adsorbent can be used in the future as a tool for removing dye pollution from water.

## Data Availability

The raw data supporting the conclusion of this article will be made available by the authors, without undue reservation.
